# Preliminary Study of Ultrasonic Welding as a Joining Process for Electrospun Nanofiber Mats

**DOI:** 10.3390/nano8100746

**Published:** 2018-09-20

**Authors:** Emilia Wirth, Lilia Sabantina, Marcus O. Weber, Karin Finsterbusch, Andrea Ehrmann

**Affiliations:** 1 Faculty of Textile and Clothing Technology, Niederrhein University of Applied Sciences, 41065 Mönchengladbach, Germany; emilia.wirth@stud.hn.de (E.W.); marcus.weber@hsnr.de (M.O.W.); karin.finsterbusch@hsnr.de (K.F.); 2 Faculty of Engineering and Mathematics, Bielefeld University of Applied Sciences, ITES, 33619 Bielefeld, Germany; lilia.sabantina@fh-bielefeld.de; 3 Departamento de Ingeniería Química, Campus de Teatinos s/n, Universidad de Málaga, Andalucía Tech, 29010 Málaga, Spain

**Keywords:** electrospinning, nanofiber mat, bonding, joining, ultrasonic welding

## Abstract

Electrospinning can be used to create nanofiber mats for diverse applications, from wound dressings and tissue engineering to filters for medical and biotechnological applications. In most of these applications, it is necessary to fix the nanofiber mat on a macroscopic textile fabric, on another nanofiber mat or within a frame to keep it at the desired position. Due to their extremely low thickness and areal mass, however, nanofiber mats are easily destroyed by sewing, and in several situations glued bonds are too thick and not flexible enough. Here we report on ultrasonic welding of polyacrylonitrile nanofiber mats, suggesting this method as a joining process without destruction of the mat morphology for thermoplastic nanofiber mats. A variety of welding patterns results in different adhesion forces between both joined nanofiber mats and different failure mechanisms, with some welding patterns enabling bonding stronger than the mats themselves. Our findings show that ultrasonic welding is a possible joining method for polyacrylonitrile nanofiber mats.

## 1. Introduction

Electrospinning enables production of nanofiber mats with fiber diameters between around ten nanometers and several micrometers. While most electrospinning instruments use a needle through which a polymer solution or melt is pressed by a syringe, needleless technologies typically place the polymer solution or melt on a wire or a rotating cylinder. In both cases, a strong electric field drags the polymer solution to an oppositely charged electrode which is shielded by a substrate on which the resulting fibers are placed, building a nanofiber mat there. In this way, nanofiber mats with a high surface: volume ratio and high porosity are created.

Such nanofiber mats can be used for a broad variety of applications, e.g., for biotechnological or medical purposes [1,2,3,4], antibacterial and antifouling surfaces [5], as catalyzers [6], or filters [7,8,9]. They can be produced from diverse biopolymers [10,11,12,13], man-made polymers [14,15,16] or blends with other materials [17,18,19].

In some of the aforementioned applications, e.g., as filters, nanofiber mats experience high loads. Due to their low thickness and areal weight (typically some g/m^2^, i.e., less than 10% of a typical printing paper), resulting in typical forces at break below 10 N [20], they must be supported by macroscopic textile fabrics or additional layers of nanofiber mats to avoid fabric failure. One idea to prevent the nanofibers from damage is using a common macroscopic yarn as the substrate in electrospinning and knitting with the resulting nanofiber-coated yarn [21]. It was found that during knitting, both sorts of fibers are mixed, resulting in a certain sheltering of the nanofibers by the macroscopic cotton fibers. Another possible method is laminating the fibers, e.g., by firstly electrospinning them and afterwards bonding them on the surface of a resin which is afterwards cured [22] or by spinning them directly on an aluminum foil on which they stick rigidly [23]. Hot-pressing a composite from a polymer membrane and polymer as well as SiO_2_ nanoparticles resulted in a significant increase of the tensile strength by melting the bond points of the nanofibers and thus linking neighboring fibers [24]. In all cases, the resulting composites cannot be used as filters anymore.

For this application, only few attempts to increase the mechanical properties without significantly changing the porous structure are reported in the literature. One approach is changing the usual plain nonwoven structure of the nanofiber mat to a honeycomb-like structure, resulting in increased filter efficiency and mechanical strength [25]. A chemical treatment can also result in increased mechanical properties of a nanofiber mat [26]. Joining the fibers at the contact points by letting solvent remain on the nanofiber mat after electrospinning can support the mechanical properties as well [27].

Lamination of a nanofiber mat on a nonwoven using a heat-press could increase the mechanical strength by more than five times, as compared to the pure nanofiber mat, while at the same time not negatively influencing the filtering properties [28]. It should be mentioned, however, that lamination pressure and temperature always influence the average pore size and thus the air and water permeability as well as possibly the hydrophobic/hydrophilic properties of a nanofiber mat [29].

Following the idea of using a heat-press nevertheless leads to another possibility which may be utilizable even for filters. By using ultrasonic welding, it is possible to bond textile fabrics or other materials along defined seam areas, while the other areas are kept in their original state. Ultrasonic welding is typically used for textile produced from man-made fibers. For PC/ABS (polycarbonate/crylonitrile butadiene styrene) blends, e.g., both materials of the blend showed different changes according to the vibrational heat during ultrasonic welding, but diffusion of ABS molecules even increased the integrity of the welded joints [30]. Besides pure thermoplastic materials, composites with carbon fibers and other materials have also been examined for ultrasonic welding [31,32]. On the other hand, non-textile thermoplastic materials [33,34] as well as metals [35,36,37] can be bonded by ultrasonic welding. 

Interestingly, even a few reports about composites in which nanofiber mats are bonded to common macroscopic textile fabrics by ultrasonic welding can be found in the literature [38,39,40], while ultrasonic welding of pure nanofiber mats to the best of our knowledge is not yet reported in the scientific literature.

Thus we report here on investigations of bonding two nanofiber mats to avoid any modifications in the filter area due to lamination or gluing processes. For this purpose, the typical textile bonding method of sewing is compared with gluing the defined seams as well as ultrasonic welding with diverse welding patterns.

## 2. Materials and Methods 

The needleless electrospinning machine “Nanospider Lab” (Elmarco Ltd., Liberec, Czech Republic) was used to create nanofiber mats on a polypropylene nonwoven as substrate which was removed before further processing. The spinning solution contained 16% polyacrylonitrile (PAN) dissolved in DMSO (min 99.9%, purchased from S3 chemicals, Bad Oeynhausen, Germany). This material was chosen since it can be electrospun from a low-toxic solvent and is nevertheless waterproof.

The spinning parameters were: high voltage 80 kV, nozzle diameter 0.8 mm, carriage speed 100 mm/s, ground-substrate distance 240 mm, electrode-substrate distance 50 mm, temperature in chamber 22 °C, relative humidity in chamber 32%. Spinning was performed for 1 h, resulting in relatively thick nanofiber mats with an areal weight of 5.4 g/m^2^.

For sewing, the double lockstitch machines Dürkopp Adler 281 (Dürkopp Adler, Bielefeld, Germany) and Juki DDL-9000C Full Digital (JUKI K. K., Tama, Japan) were used with a needle gauge of 80 Nm and a stitch length of 3.5 mm (Dürkopp Adler) and 2.8 mm (Juki), respectively. The first machine was equipped with a Teflon presser foot to avoid undesired sticking of nanofiber layers.

The following adhesives were used for the gluing tests: The textile glue Gütermann HT2 (Gütermann GmbH, Gutach im Breisgau, Germany) and the thermoplastic polyurethane adhesive film Pepinoplast 4450 (Pepin Production GmbH, Wuppertal, Germany). The ironing system Veit Varioset S + B/180 (Veit GmbH, Vlotho, Germany) was applied for ironing the adhesive film for 5 s at 180 °C without steam. This temperature is below typical stabilization temperatures for PAN and can thus be expected not to influence the nanofiber morphology or the chemical composition of the PAN nanofibers.

**Figure 1:**
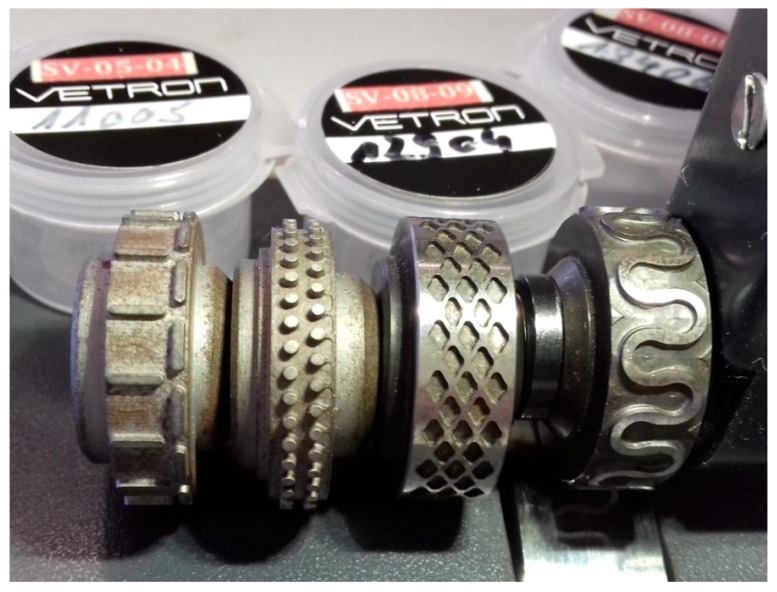
Anvil wheels chosen for this study. From left to right: T-stitch, point 2-rowed, diamond, and omega.

Ultrasonic welding was performed with a flatbed ultrasonic welding machine Vetron 5064 (VETRON TYPICAL Europe GmbH, Kaiserslautern, Germany) with an ultrasonic frequency of 35 kHz and a welding seam width of 13 mm maximum, using the following patterns of anvil wheels: Diamond (W 12904), omega (W 13406), point 2-rowed (W 11005) and T-stitch welding (W 13013). Figure 1 depicts the four chosen patterns. The welding power (0–1000 W) and the pressure (0–150 bar) were varied. It should be mentioned that the pressures achievable here are typical for the textile industry, but of the same order of magnitude as pressures reported for ultrasonic welding of metals (e.g., 14 bar-32 bar [41], 72 bar [42]).

It should be mentioned that during ultrasonic welding, high temperatures (around several hundred degree Celsius) can be expected to be reached for short times. This means that examination of the ultrasonically welded area is necessary to investigate whether the chemical composition of the PAN fibers is modified by a stabilization process (typically between approx. 210 °C and 300 °C) or whether the PAN is degraded or pyrolized at even higher temperatures.

For the optical examination of the bonds, a confocal laser scanning microscope (CLSM) VK-100 with a nominal magnification of 2000× and a digital optical microscope VHX-600 (both from Keyence, Osaka, Japan) were used. Mechanical examination of the seams was performed with a Zwick Roell 1455 universal testing machine using a 0.2 N–100 N force transducer according to ISO 13935-2:2014 for the determination of the maximum force to seam rupture. 

## 3. Results and Discussion

### 3.1. Sewing

**Figure 2:**
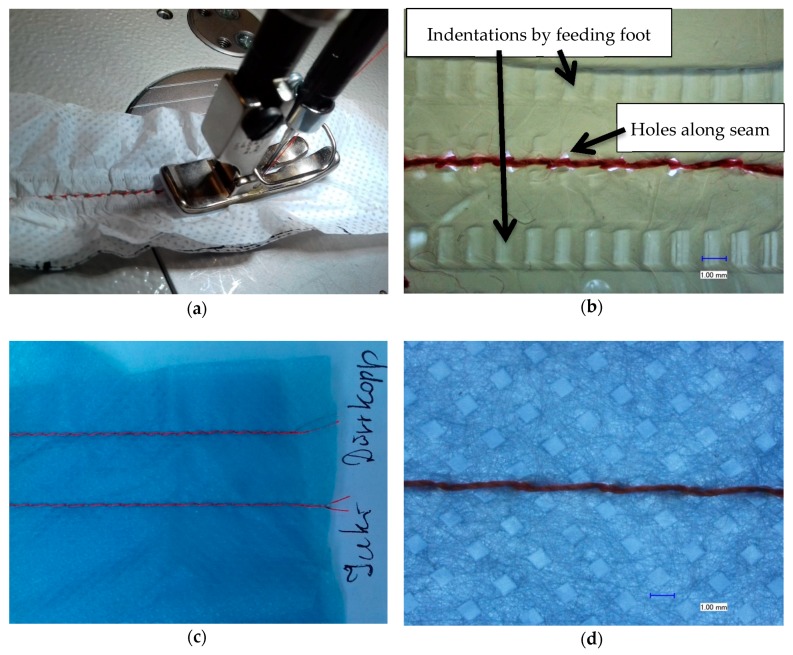
Examples of the sewing process: (**a**) Macroscopic depiction of the sewn nanofiber mats during sewing with Juki DDL-9000C Full Digital sewing machine; (**b**) Microscopic image of the seam and its environment; (**c**) Macroscopic depiction of the sewn substrate nonwoven; (**d**) microscopic image of a seam in the substrate nonwoven.

Figure 2a shows the sewing process of two nanofiber mats and Figure 2b shows a microscopic image of the result of sewing. The pattern of small squares stems from the substrate used during electrospinning (cf. Figure 2d). Directly during sewing, the problems with this process become visible: The transport of the fabric does not work properly, but creases the nanofiber mats due to their small thickness and their electrostatic charge. The microscopic image depicts this issue in more detail: On the one hand, each stitch destroys the nanofiber mats; on the other hand, the feeding foot also leaves deep marks in the nanofiber mats. Many parameters are responsible for the optimum quality of the sewing seam. The optimum settings of sewing machine parameters such as under thread and upper thread tension, stitch length, foot shear, correct sewing thread selection, sewing needle size and needle shape as well as the quality of the material used play an important role. The needle shape is also decisive for the quality of the sewing seam. Usually a strongly rounded ball tip is used for fine materials, displacing the material threads at certain points and not destroying them. Among other parameters, the thread thickness, the material properties such as surface quality, thickness and density have an enormous influence on the quality of the seam. Finding out the optimum parameter for processing very fine materials such as nanofiber mats could help here, but these settings also have limits.

As Figure 2c,d show, sewing macroscopic materials like the nonwoven used as a substrate in electrospinning is not problematic. Here, no marks of the feeding foot are visible nor undesired crinkling of the seam (Figure 2c). In the microscopic image, it becomes clear that the nonwoven is not destroyed by the needle, but the fibers are pushed aside by the rounded tip of the needle (Figure 2d). 

Obviously, this typical textile joining process is not suitable for nanofiber mats, although here machines for underwear were used instead of the coarser machines for common garments.

### 3.2. Gluing

Gluing was on the one hand performed using commercially available textile glue and on the other hand with an adhesive film which is also common in the textile industry. 

**Figure 3:**
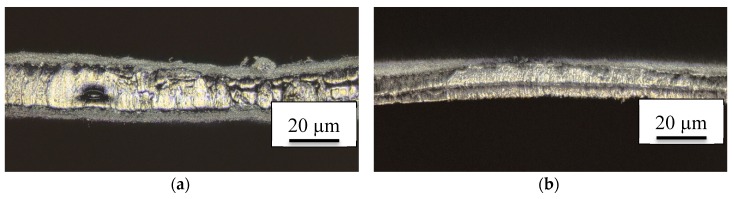
Nanofiber mats joined with textile glue: (**a**) Drying without pressure; (**b**) Drying under pressure.

Figure 3 depicts the results of joining two nanofiber mats with the textile glue, dried at the air without any additional treatment or with a constant weight on the whole seam, resulting in a pressure of 300 Pa. In both cases, the gluing layer is visibly thicker than the nanofiber mats. This method does not seem to be suitable to create bonds which do not alter the physical properties of the nanofiber mats too severely.

**Figure 4:**
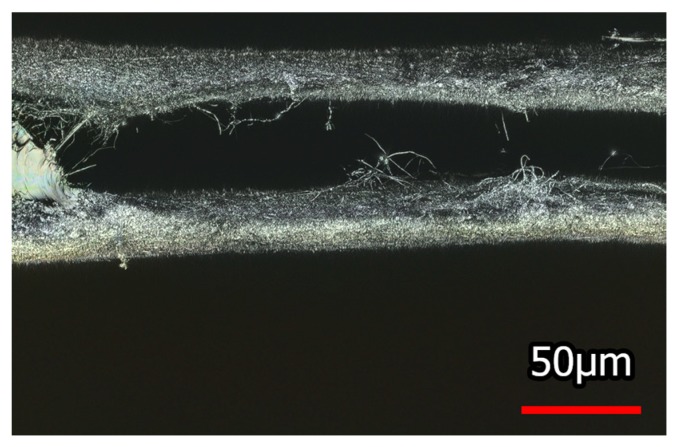
Nanofiber mats joined with thermoplastic polyurethane adhesive film.

Similarly, samples were bonded with the thermoplastic polyurethane adhesive film. One exemplary cross-section is depicted in Figure 4. It is clearly visible that the seam is not continuously connected to both nanofiber mats. In addition, the seam is again quite thick. Nevertheless, samples bonded with thermoplastic polyurethane adhesive film will be investigated with respect to their mechanical properties since this method is very common in the textile industry and does not necessitate unusual equipment.

### 3.3. Ultrasonic Welding

In ultrasonic welding, different parameters can be varied, especially the anvil wheel–defining the shape of the connected area along the seam–, the pressure and the power. To explain these parameters, their influence is depicted in the next figures.

It should be mentioned that the anvil wheel patterns chosen here were chosen according to the following ideas: Firstly, preliminary tests with a single fine welding line of a cutting wheel W20001 showed no adhesion at all; thus all very thin connections were ignored. Second, it can be assumed to be interesting to investigate the influence of connected vs. disconnected patterns. Here, we chose the point 2-rowed as an example for a disconnected pattern, and the T-stitch as a transition to connected patterns. Amongst the connected patterns, differences can be expected between rounded shapes– here represented by the omega pattern–and angular patterns with sharp changes of the directions of the connection lines, here represented by the diamond pattern. Unfortunately this leads to the effect that the welded areas are not always the same, an influence which has to be kept in mind in the discussion of the results.

**Figure 5:**
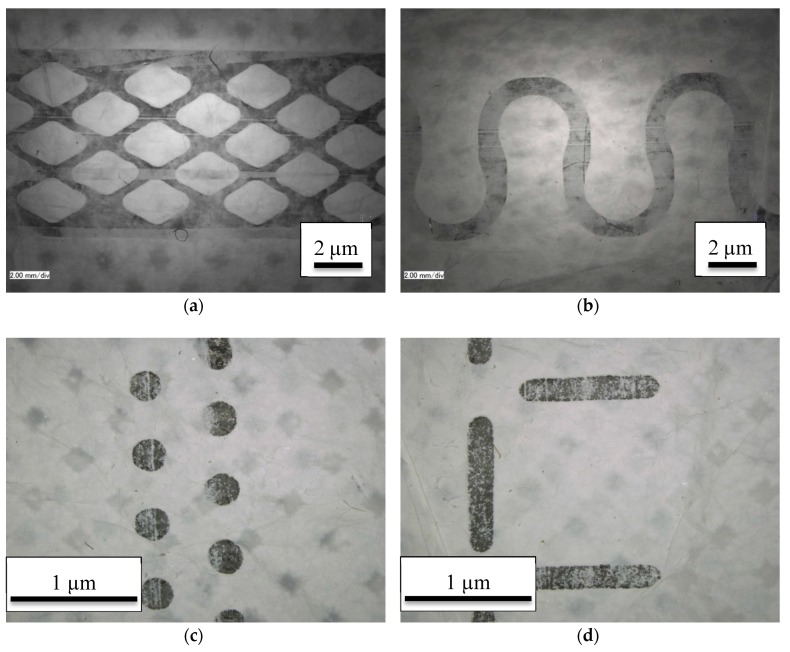
Microscopic images of the ultrasonic welding seams prepared with a pressure of 65% = 97.5 bar, a power of 750 W and different anvil wheels: (**a**) diamond (W 12904); (**b**) omega (W 13406); (**c**) point 2-rowed (W 11005); (**d**) T-stitch welding (W 13013). The scales differ due to the different seam widths.

Figure 5 shows the patterns of different anvil wheels used in this study on nanofiber mats bonded by ultrasonic welding. The different patterns and correspondingly different percentages of the seam widths as well as the varying seem widths are clearly visible. This suggests possible differences in the adhesion between both nanofiber mats.

To enable easier comparison, the welded areas were calculated for all patterns, resulting in a nominal seam width which is defined as the width of a linear seam with a welding area identical to the sum of the areas welded by the patterns used here, averaged along the seam. This results in a nominal seam width of 3.53 mm (diamond), 2.94 mm (omega), 0.21 mm (point 2-rowed) and 0.31 mm (T-stitch), respectively. It must be underlined that both connected patterns–diamond and omega–result in approx. ten times larger nominal seam widths than both disconnected patterns–point 2-rowed and T-stitch–, clearly showing that both parameters cannot be examined independently with the available anvil wheels.

This, however, is still only half the truth. Since not the whole welded area is examined at the same time, but only a single line has to withstand the applied force, the more important parameter is the relative bonded area, i.e., the fraction of the welding area which is connected. More precisely, broader seam patterns will show larger adhesion forces in a shear test, but not in a seam rupture test according to ISO 13935-2:2014. The averaged relative welded areas are 46% (diamond), 44% (omega), 25% (point 2-rowed) and 20% (T-stitch), showing much smaller deviations between the different patterns, but still two sets of patterns with larger and smaller percentage welded area. Both these calculation approaches suggest that diamond and omega should result in significantly higher seam rupture strengths than point 2-rowed and T-stich patterns.

Another important factor is the pressure exerted on the wheel. On the one hand, the pressures below approx. 60% (90 bar) and above approx. 80% (120 bar) show a clearly increased risk of fabric damage along the seam. The usable pressure range is thus above typical pressures used for ultrasonic welding of metals. On the other hand, the pressure is crucial for the adhesion properties.

**Figure 6:**
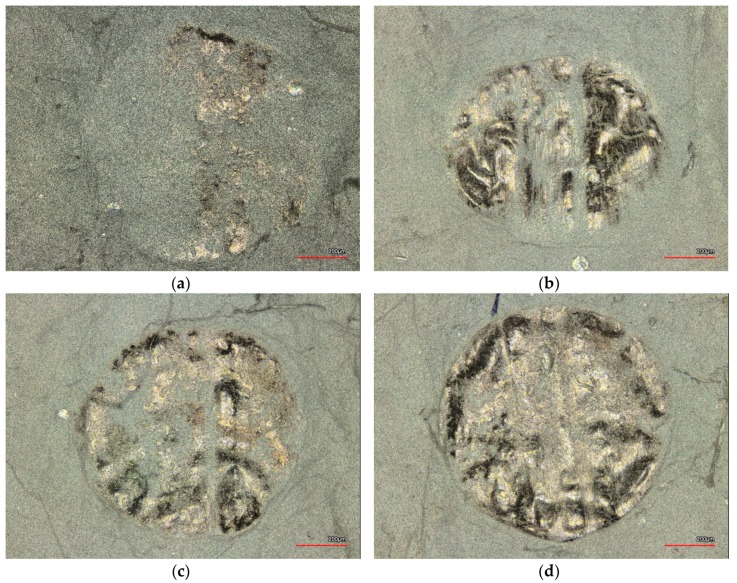
Confocal laser scanning microscope (CLSM) images of the ultrasonic welding seams prepared with point 2-rowed anvil wheel, a power of 750 W and different pressures: (**a**) 50% = 75 bar; (**b**) 60% = 90 bar; (**c**) 70% =105 bar; (**d**) 90% = 135 bar.

Figure 6 shows a series of ultrasonic welding dots, prepared with the point 2-rowed anvil wheel under different pressures between 50% (75 bar) and 90% of the maximum pressure (135 bar). Since PAN changes its color during stabilization at temperatures higher than approx. 200 °C [23], the areas which were mostly heated due to the ultrasonic welding process can easily be recognized. It is clearly visible that for the lowest pressure of 50%, not much change is induced in the upper fabric’s morphology, while for the highest pressure applied here of 90%, nearly the whole area of the dot has changed its color. It must be mentioned that it is also clearly visible that the fabric seems to be wrinkled in the dot area, apparently due to the short-time heat stress which seems to result in a relaxation of the material inside this area.

**Figure 7:**
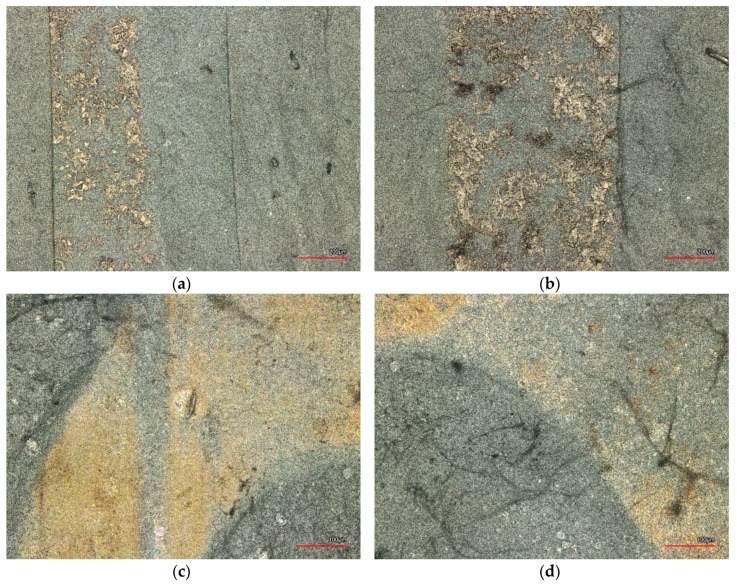
CLSM images of the ultrasonic welding seams prepared with different anvil wheels and pressures: (**a**) T-stitch welding with pressure 60% = 90 bar and 600 W; (**b**) T-stitch welding with pressure 70% = 105 bar and 700 W; (**c**) omega welding with pressure 65% = 97.5 bar and 750 W; (**d**) omega welding with pressure 65% = 97.5 bar and 750 W at another position. The scales differ due to the different seam widths.

This is why the other anvil wheels with larger connected areas were also tested. Some of the results are depicted in Figure 7. Comparing Figure 7a,b, it can be recognized that for the slightly lower pressure of 60% (Figure 7a), only half of the planned seam width has changed its color, which has changed for a pressure of 70%. Nevertheless, the ratio of stabilized seam area is still low.

This changes, however, for the omega-shaped anvil wheel. Using this continuous shape, relatively large areas of the actual seem have changed their color in Figure 7c. It must nevertheless be mentioned that even with this anvil wheel, still areas exist which are significantly worse bonded, as can be seen by the strongly reduced color change (Figure 7d). The same results were found with the second anvil wheel creating a continuous seam, i.e., the diamond-shaped one.

Generally, both continuous shape–omega and diamond–showed more regular results. Microscopic examinations nevertheless revealed significant deviations between different areas which can be attributed to creases in the nanofiber mats, resulting in an uneven surface and thus differing slit widths along the seam. For all shapes, a pressure of approx. 65% was found to be ideal with respect to the first optical examination and the error rate. Errors occurred especially for both the continuous shapes, omega and diamond, for smaller or larger seam pressures, while both other patterns could also be produced with higher or lower pressures. 

**Figure 8:**
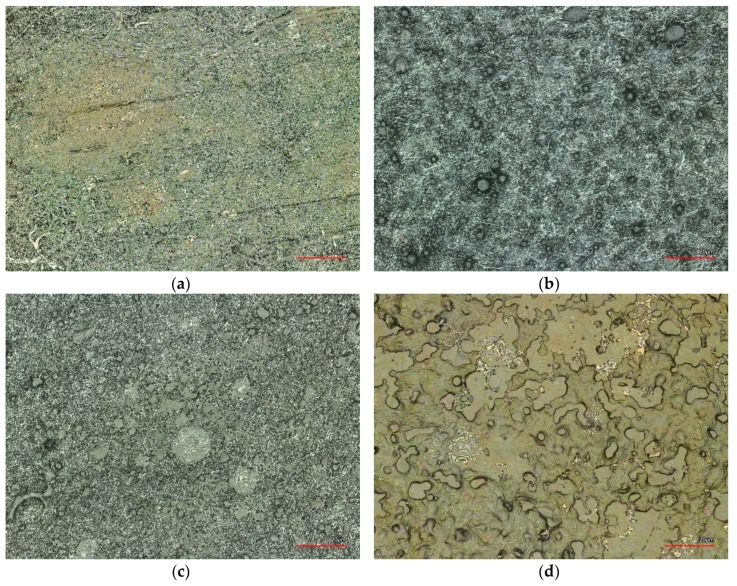
CLSM images of (**a**) an ultrasonic welding seam prepared with the omega pattern; PAN nanofiber mats pressed at different temperatures for 1 min at 1.2 bar: (**b**) 200 °C; (**c**) 220 °C; (**d**) 250 °C. All scale bars show 20 µm.

Finally, the influence of the short temperature pulse due to the ultrasonic welding process is further investigated. Figure 7 has already suggested that typically temperatures above 200 °C are reached since these temperatures are known to stabilize the PAN nanofiber mat [23]. Since time-resolved measurements of the maximum temperatures are complicated to perform, Figure 8 depicts images of long-term heat-pressing two PAN nanofiber mats instead for comparison. Additionally, Figure 8a shows a typical CLSM image of the ultrasonic welded seam produced with the omega pattern. Here it is again visible that only parts of the welding area have changed their color, indicating that the areas which are still grey did not reach as high temperatures.

Comparing the colors visible here with those gained by pressing two PAN nanofiber mats at different temperatures (200–250 °C, cf. Figure 8b–d) indicates that the highest temperatures reached during ultrasonic welding are in the range of 250 °C; higher temperatures result in much darker nanofiber mats [23]. It must be mentioned that in contrast with the pressure tests, the welded seam still shows mostly fibrous areas with some membrane-like areas where the highest temperatures are reached. This the opposite result to the nanofiber mats pressed at 250 °C, which have completely lost their original morphology. This finding might lead to the idea that using a heated press may be advantageous to create reliable seams, as compared to ultrasonic welding. This, however, does not work. Due to the very small pressure which is typical for textile heat presses, both nanofiber mats pressed in the heat press can be separated easily.

Mechanical examinations of the ultrasonic welding seams are depicted in the next sections.

### 3.4. Adhesion Tests With Glued Seams

Seams glued with Pepinoplast were 6 cm wide and 1 cm long. This seam width was chosen to average over a relatively broad area and thus over possible deviations in the bond strength which could be expected due to the examination of the cross-sections (Figure 4). Experiments were performed on 5 nominally identical samples.

In all seam rupture tests, the seam itself with the additional Pepinoplast layer between both nanofiber mats was found to be stronger than the nanofiber mats themselves, i.e., failure always occurred along one of the edges of the seam. The average maximum force was (1.3 ± 0.6) N, showing the broad spectrum of tensile strengths of the nanofiber mats themselves. This can also be regarded as a benchmark for the investigation of other bonding methods–ideally, they should result in maximum forces to seam rupture of approx. 2 N for a seam width of 6 cm. 

### 3.5. Adhesion Tests with Ultrasonically Welded Seams

**Figure 9:**
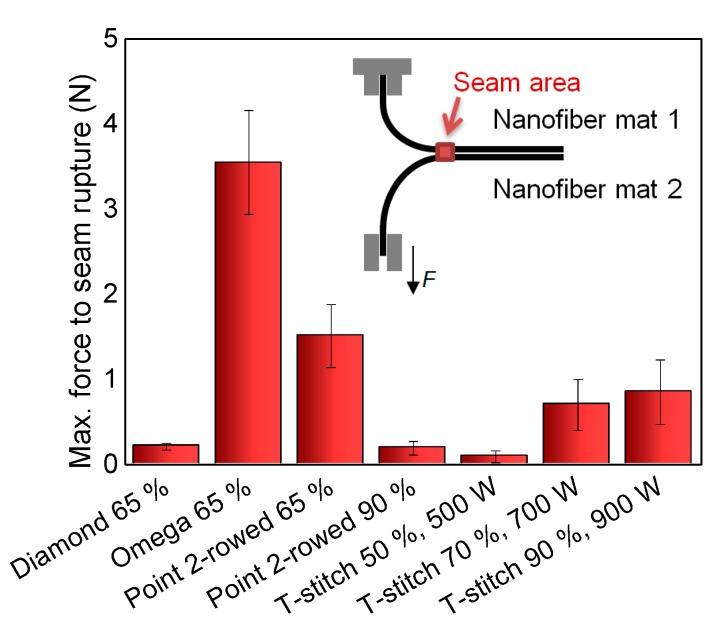
Maximum forces to seam rupture, measured for different ultrasonic welding patterns and pressures. Inset: measurement principle.

Next, the seam strength of ultrasonically welded seams was investigated. Only those combinations of the aforementioned anvil wheel patterns and pressures were examined which could be created reliably, i.e., with less than 10% errors (broken nanofiber mats along the seam) during ultrasonic welding, as described above. The results are depicted in Figure 9; the measurement principle is depicted in the inset. 

Unexpectedly, the diamond pattern–with the largest absolute and relative welded area, as calculated before–necessitates only very small maximum forces. This can be attributed to the very thin welding lines throughout most of the pattern and the sharp corners, opposite to the omega shape. Apparently, thicker lines and rounded connections are advantageous.

The largest forces to seam rupture were found for the omega pattern, used with a pressure of 65%, and the point 2-rowed pattern, also with a pressure of 65%. These pressures correspond to the values for which the least errors occurred during welding. Using the point 2-rowed pattern with a higher pressure of 90%, the maximum force to seam rupture is significantly decreased. This may be correlated with the increased wrinkling of the welding points which was visible in Figure 6, possibly partly destroying the connected material and thus also the bond.

For the T-stitch pattern, three pressures were compared. Here, the higher pressures resulted in significantly increased maximum forces to seam rupture, as compared to the lowest pressure. This is easily understandable by the results depicted in Figure 7, showing that even for a pressure of 60%, only parts of the actual seam are really connected.

These results show that while most ultrasonic welding seams have too low maximum forces to seam rupture to be used for reliable connections of nanofiber mats, carefully choosing the optimum combination of anvil wheel–i.e., welding pattern–and pressure on the nanofiber mats may result in sufficient seam strengths. Nevertheless it must be mentioned that further optimization is necessary, not only with respect to the welding process itself but also to the preparation of the nanofiber mats which shall be connected, especially to avoid wrinkles which cause deviations in the seam strength.

## 4. Conclusions

In a first-principle study, we have shown that ultrasonic welding can be used to join thermoplastic nanofiber mats produced by electrospinning. While glued seams are often stronger, ultrasonically welded connections have the advantages of avoiding thick places as well as areas with higher bending stiffness. Future tests are necessary to optimize the ultrasonic welding process as well as the preparation of the nanofiber mats before joining them. In addition, different joining methods should be compared in real applications, such as filters in liquid environments.
